# Variations in volume of emergency surgeries and emergency department access at a third level hospital in Milan, Lombardy, during the COVID-19 outbreak

**DOI:** 10.1186/s12873-021-00445-z

**Published:** 2021-05-10

**Authors:** Laura Castoldi, Monica Solbiati, Giorgio Costantino, Elena Casiraghi

**Affiliations:** 1 Fondazione IRCCS Ca’ Granda Ospedale Maggiore Policlinico, UOSD Chirurgia d’Urgenza,, Milano, Italy; 2 Fondazione IRCCS Ca’ Granda Ospedale Maggiore Policlinico, UOC Pronto Soccorso e Medicina d’Urgenza, Milan, Italy; 3 Dipartimento di Scienze Cliniche e di Comunità, Università degli Studi di Milano, Milan, Italy; 4 Anacleto Lab, Computer Science Department, Università degli Studi di Milano, Milan, Italy; 5 MIPS Lab, Computer Science Department, Università degli Studi di Milano, Milan, Italy; 6 CINI-AIIS, Italian National Laboratory in Artificial Intelligence and Intelligent Systems, University of Modena and Reggio Emilia, Modena, Italy

**Keywords:** Coronavirus, COVID-19, Emergency surgery, Emergency department attendance, Emergency department overcrowding

## Abstract

**Background:**

During the recent outbreak of COVID-19 (coronavirus disease 2019), Lombardy was the most affected region in Italy, with 87,000 patients and 15,876 deaths up to May 26, 2020. Since February 22, 2020, well before the Government declared a state of emergency, there was a huge reduction in the number of emergency surgeries performed at hospitals in Lombardy. A general decrease in attendance at emergency departments (EDs) was also observed. The aim of our study is to report the experience of the ED of a third-level hospital in downtown Milan, Lombardy, and provide possible explanations for the observed phenomena.

**Methods:**

This retrospective, observational study assessed the volume of emergency surgeries and attendance at an ED during the course of the pandemic, i.e. immediately before, during and after a progressive community lockdown in response to the COVID-19 pandemic. These data were compared with data from the same time periods in 2019. The results are presented as means, standard error (SE), and 95% studentized confidence intervals (CI). The Wilcoxon rank signed test at a 0.05 significance level was used to assess differences in per-day ED access distributions.

**Results:**

Compared to 2019, a significant overall drop in emergency surgeries (60%, *p* < 0.002) and in ED admittance (66%, p ≅ 0) was observed in 2020. In particular, there were significant decreases in medical (40%), surgical (74%), specialist (ophthalmology, otolaryngology, traumatology, and urology) (92%), and psychiatric (60%) cases. ED admittance due to domestic violence (59%) and individuals who left the ED without being seen (76%) also decreased. Conversely, the number of deaths increased by 196%.

**Conclusions:**

During the COVID-19 outbreak the volume of urgent surgeries and patients accessing our ED dropped. Currently, it is not known if mortality of people who did not seek care increased during the pandemic. Further studies are needed to understand if such reductions during the COVID-19 pandemic will result in a rebound of patients left untreated or in unwanted consequences for population health.

**Supplementary Information:**

The online version contains supplementary material available at 10.1186/s12873-021-00445-z.

## Background

The outbreak of COVID-19 started in Wuhan, China, in December 2019 and quickly spread beyond the borders of the People’s Republic of China. The World Health Organization (WHO) declared COVID-19 a worldwide pandemic on March 11, 2020 when more than 118,000 people were affected by severe acute respiratory syndrome-related coronavirus-2 (SARS-CoV-2) throughout the world. In Italy, the first patient who tested positive for SARS-CoV-2 was admitted to the intensive care unit (ICU) of Codogno Hospital, located in a town near Lodi (Lombardy), on February 20, 2020. On February 22, the Government of Lombardy instituted a task force to address the emergency and established containment measures by quarantining several towns near Codogno (so-called “red zones”), where several COVID-19 clusters had emerged. Within fourteen days, ICU admissions exceeded 550 and hospital admissions totalled 2217 [1]. Due to the rapid spread of infection, on March 8 Lombardy was quarantined and self-isolation measures were instituted to slow virus transmission. As of April 20, more than 19,000 COVID-19 patients were admitted to Lombardy hospitals [2].

Of note, on March 8 the Lombardy Government established the “Hub and Spoke” model. Accordingly, certain hospitals were designated as hubs for healthcare time-dependent diseases, i.e. polytrauma, and cardiovascular and neurological emergencies [3]. Our hospital, IRCCS Foundation Cà Granda Ospedale Maggiore Policlinico, which is a 900-bed university hospital in downtown Milan, was temporarily not allowed to admit polytrauma patients. However, emergency rooms, emergency operating areas (according to subsequent recommendations of international surgical societies) [4–6], a surgical non-intensive care ward, and ICU beds for non-COVID patients were set up, in addition to areas for infectious or suspected COVID-19 patients.

Since February 22, the Policlinico Hospital emergency department has prepared for COVID-19 patients with logistic measures and new triage rules. All elective surgeries were cancelled, and surgeons were reallocated to ED and COVID-19 wards to provide care for less critical patients. At the same time, a drop in ED attendance for non-COVID-19 diseases, especially patients with surgical complaints, was observed. This reduction inversely followed an increase in COVID-19 patients.

The aim of this paper is to quantify the extent of these observed reductions, to assess the characteristics of the potentially surgical patients who did not access to ED, and to analyze how surgical emergencies could have been managed without ED access.

## Methods

### Study design

A retrospective, observational study was performed to assess all the emergency surgeries performed and the attendance number at the ED of a level III university hospital immediately before, during and after a progressive community lockdown in response to the COVID-19 pandemic. By using an anonymous hospital-based administrative database, automatically generated with data from ED software (PSNet, Hitech SpA, Software Engineering), the data on ED attendances were collected and analyzed along four time periods:

- ° Period 1 (February 21–March 8; 16 days in 2020, due to it being a leap year; 15 days in 2019), when the first COVID-19 patient was admitted to Codogno Hospital and ten small towns near Milan were quarantined.
- ° Period 2 (March 9–21; 14 days), start of the lockdown in Lombardy, as well as in 14 provinces in Piemonte, Veneto, Emilia Romagna, and Marche.
- ° Period 3 (March 22–April 21; 32 days), when lockdown was expanded to include the entire Italian nation. All non-necessary businesses and industries were shut down.
- ° Period 4 (April 22–May 12; 19 days), when ED attendance numbers for COVID-19 patients decreased and it was clear that the national lockdown would have been attenuated, as it was effectively announced on April 26 (the beginning of the so-called “Phase 2”).

For each of these four periods, attendances at the ED were stratified according to specialties: general medicine, surgery, specialist examinations (ophthalmology, otolaryngology, traumatology, and urology), psychiatric examinations and attendances for domestic violence. Number of deaths in the ED and numbers of patients who left the ED without being seen by a physician (LWBS) were also collected. All of these data were compared with the same time periods in 2019.

Similarly, emergency surgical interventions during the same time periods in 2020 and in 2019 were compared.

### Statistical analysis

Data are presented as means, standard errors (SE), and 95% studentized confidence intervals (CI). All the 95% studentized confidence intervals were computed with 2500 bootstrap iterations.

The Wilcoxon rank signed test at a 0.05 significance level was used to assess the differences in the per-day access distribution according to the following groups: medicine, surgery, specialist examinations, psychiatry, domestic violence, LWBS, and deaths.

For each category, drop percentages of the number of events in 2020 (x_2020_) with respect to the number of events in 2019 (x_2019_) were computed as follows: drop% = abs(x_2019_-x_2020_)/ x_2020_.

Matlab software (The MathWorks, Inc., Natick, MA, USA) was used to perform data analysis and to generate plots.

## Results

**Fig. 1:**
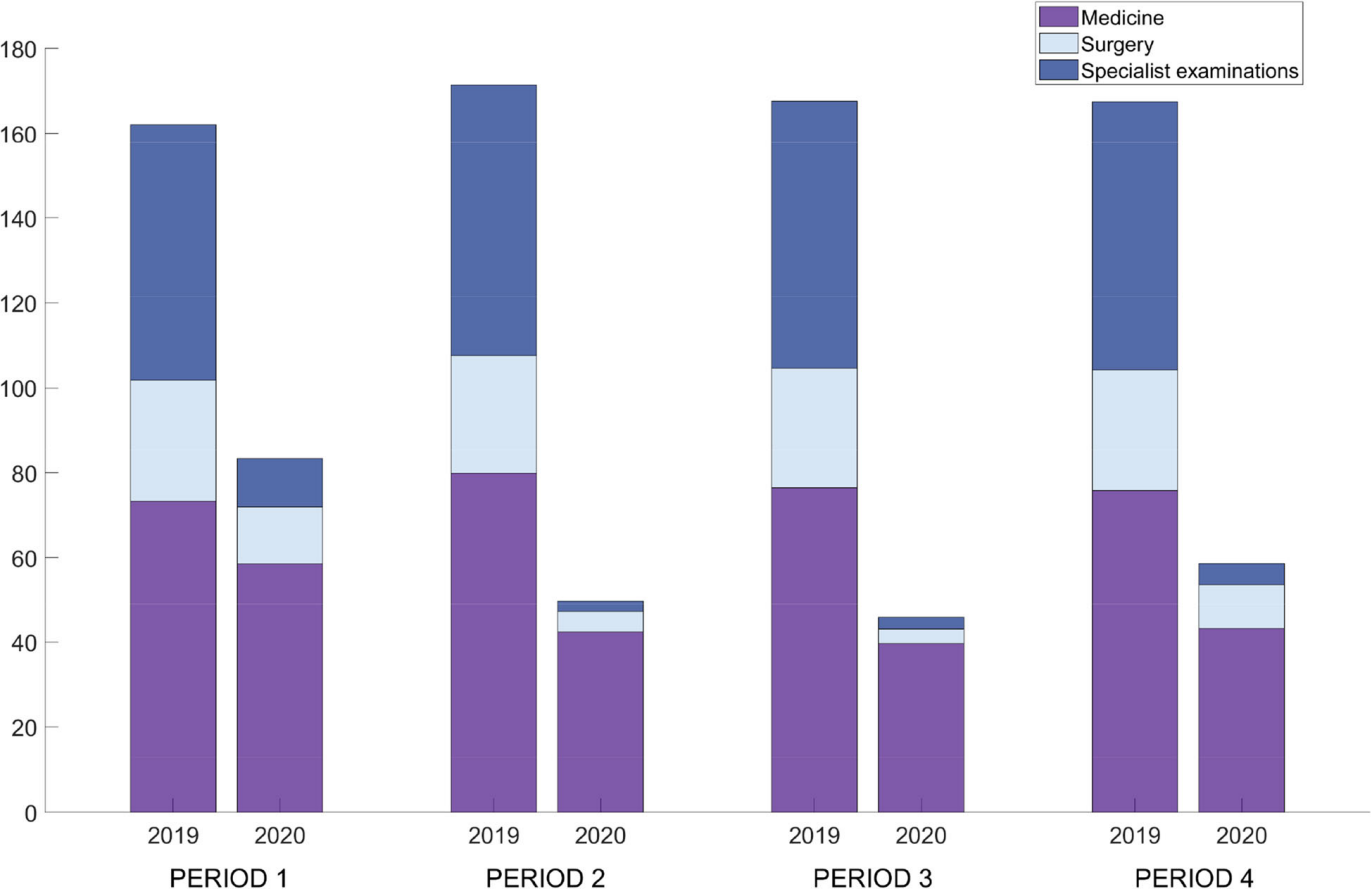
Per-period mean daily ED accesses for medicine, surgery and specialist examinations in 2019 and 2020

Compared to the same periods in 2019, a 48% reduction in overall ED attendances was observed for non-COVID-19 diseases starting February 22, 2020 (Period 1) (3108 vs 1607). Figure 1 shows the daily counts of accesses in 2020 compared to 2019.

This trend continued through second and third periods, with a 61% drop in total admittances (5307 ED attendances in 2020 vs 15,464 in 2019) **(**Table 1**)**.

**Table 1:** Mean daily access ± standard error, and the 95% bootstrap Studentized confidence interval, for each year and each category

Period		1		2		3		4		Total	
		Daily accesses (mean ± SE)	95% CI	Daily accesses (mean ± SE)	95% CI	Daily accesses (mean ± SE)	95% CI	Daily accesses (mean ± SE)	95% CI	Daily accesses (mean ± SE)	95% CI
Medicine	2019	78 ± 1.86	[73, 82]	79 ± 1.37	[76, 82]	76 ± 1.58	[73, 80]	76 ± 2.52	[69, 80]	77 ± 0.95	[75, 79]
	2020	62 ± 2.55	[58, 71]	42 ± 1.68	[39, 46]	40 ± 1.68	[37, 43]	45 ± 2	[41, 49]	46 ± 1.35	[43, 49]
	**% drop**	21 ± 3.35	[7, 26]	47 ± 0.86	[44, 48]	49 ± 1.32	[45, 51]	40 ± 0.8	[39, 42]	40 ± 1.34	[38, 43]
Surgery	2019	30 ± 1.49	[27, 33]	28 ± 1.18	[25, 31]	28 ± 0.95	[26, 30]	28 ± 1.25	[26, 32]	29 ± 0.59	[27, 30]
	2020	14 ± 1.88	[8, 17]	5 ± 0.59	[3, 6]	3 ± 0.35	[3, 4]	11 ± 0.76	[9, 12]	8 ± 0.66	[6, 9]
	**% drop**	56 ± 2.51	[51, 62]	85 ± 0.7	[83, 86]	88 ± 0.53	[87, 89]	63 ± 1.45	[58, 65]	74 ± 1.61	[71, 78]
Spec. examinations	2019	64 ± 2.4	[59, 71]	65 ± 1.97	[61, 69]	63 ± 2.62	[58, 68]	62 ± 3.55	[53, 69]	63 ± 1.41	[61, 66]
	2020	12 ± 1.74	[8, 15]	3 ± 0.51	[1, 4]	3 ± 0.31	[2, 3]	5 ± 0.5	[4, 6]	5 ± 0.55	[4, 6]
	**% drop**	81 ± 2.33	[76, 87]	96 ± 0.35	[95, 97]	96 ± 0.28	[95, 96]	92 ± 0.58	[91, 93]	92 ± 0.77	[91, 94]
Psychiatry	2019	5 ± 0.44	[4, 5]	4 ± 0.44	[3, 5]	4 ± 0.38	[4, 5]	5 ± 0.67	[3, 6]	4 ± 0.24	[4, 5]
	2020	3 ± 0.37	[2, 4]	2 ± 0.37	[1, 2]	1 ± 0.2	[1]	2 ± 0.31	[1, 3]	2 ± 0.16	[1, 2]
	**% drop**	34 ± 7.03	[20, 52]	50 ± 4.11	[43, 69]	74 ± 2.46	[69, 79]	65 ± 3.83	[54, 71]	60 ± 2.38	[56, 66]
Domestic Violence	2019	**29** 1.93 ± 0.29	[1.1, 2.47]	**36** 2.57 ± 0.6	[0.52, 3.61]	**51** 1.70 ± 0.26	[0.83, 2.21]	**24** 1.33 ± 0.26	[0.36, 1.68]	**140** 1.75 ± 0.17	[1.34, 2.18]
	2020	**22** 1.38 ± 0.27	[0.75, 2.04]	**5** 0.36 ± 0.16	[0.05, 0.61]	**8** 0.27 ± 0.09	[0.04, 0.45]	**22** 1.22 ± 0.26	[0.51, 1.52]	**57** 0.70 ± 0.11	[0.54, 0.86]
	**% drop**	**24** −160 ± 183.04	[−427.48, 4673.33]	**90** 86 ± 2.04	[82.41, 91.2]	**80** 85 ± 1.66	[80.96, 89.48]	**10** 10 ± 8.92	[− 10.19, 31.67]	**60** 55 ± 4.38	[47.45, 62]
Deaths	2019	**4** 0.27 ± 0.11	[0, 0.41]	**5** 0.36 ± 0.16	[0.01, 0.59]	**8** 0.27 ± 0.09	[0, 0.40]	**6** 0.33 ± 0.13	[0, 0.48]	**23** 0.29 ± 0.06	[0.04, 0.46]
	2020	**6** 0 .38 ± 0.15	[0, 0.62]	**19** 1.36 ± 0.32	[0.54, 2.02]	**27** 0.90 ± 0.16	[0.54, 1.06]	**16** 0.89 ± 0.18	[0.44, 1.29]	**68** 0.84 ± 0.1	[0.57, 1.02]
	**% raise**	**50** 89 ± 50.37	[− 239.76, 167.64]	**280** 348 ± 78.77	[187.93, 522.51]	**238** 332 ± 48.04	[196.93, 428.47]	**167** 248 ± 55.27	[106.99, 360.31]	**196** 259 ± 26.77	[172.36, 311.65]

Since 2020 is a leap year, we averaged the accesses on February 28, 2019 and February 29, 2020, so that for both the years the whole period contains 80 days

With regard the Domestic Violence and the Deaths categories, due to few events, we report both the number of events in the whole period (**bold character)**, and the mean daily events (and their CI). Similarly, we computed the percentage variation both by using the whole number of events and by averaging the daily raise percentages (where values equals to zero for days in 2019 were substituted by the value 0.1 to avoid division by zero)

Admittance for surgery also had mean decreases of 56, 85 and 88% through Periods 1, 2, and 3, respectively. Compared with 2019, a 74% reduction was observed over all the four periods in 2020 (2313 vs 609).

During Periods 1, 2, 3 and 4 emergency surgery was performed in 24 patients, compared to 60 cases in 2019, with a statistically significant decrease of 60% (*p* < 0.001). Categorizing the surgeries performed according to indications and applying the Wilcoxon sided test, significant differences in the distributions of appendicitis (8 vs 20, *p <* 0.001) and incarcerated hernia (1 vs 10, *p <* 0.001) were observed (Table 2).

**Table 2:** Absolute number of surgeries performed in 2019 and 2020, clustered for disease

Disease	2019					2020					***p*** value
	Period 1	Period 2	Period 3	Period 4	Total	Period 1	Period 2	Period 3	Period 4	Total	
Appendicitis	4	1	9	6	20	0	0	4	4	8	< 0.001
Cholecystitis	0	0	2	0	2	1	0	1	0	2	n.s.
Diverticulitis	1	0	1	0	2	0	0	0	0	0	n.s.
Intestinal perforation (non diverticulitis)	2	1	0	1	4	0	0	2	0	2	n.s.
Incarcerated inguinal hernia/ Incarcerated umbilical or postoperative hernia	0	2	5	3	10	1	0	0	0	1	< 0.001
Gastro-duodenal perforation/hemorrhage	2	0	0	2	4	0	1	0	1	2	n.s.
Small bowel obstruction	1	1	4	3	9	0	1	2	3	6	n.s.
Colon obstruction	1	1	2	0	4	0	0	1	0	1	n.s.
Mesenteric ischemia	0	0	1	1	2	0	0	0	0	0	n.s.
Abscess	0	1	0	2	3	0	0	1	1	2	n.s.
**TOTAL**	11	7	24	18	**60**	2	2	11	9	**24**	**< 0.002**

*n.s* non significant

For general medicine accesses, the drop was 21%, 47 and 49% in Periods 1–3. Also specialist examinations showed an impressive drop: 81% in Period 1, and 96% in Periods 2 and 3, with only a small drop reduction in period 4 (92% reduction compared to 2019).

**Fig. 2:**
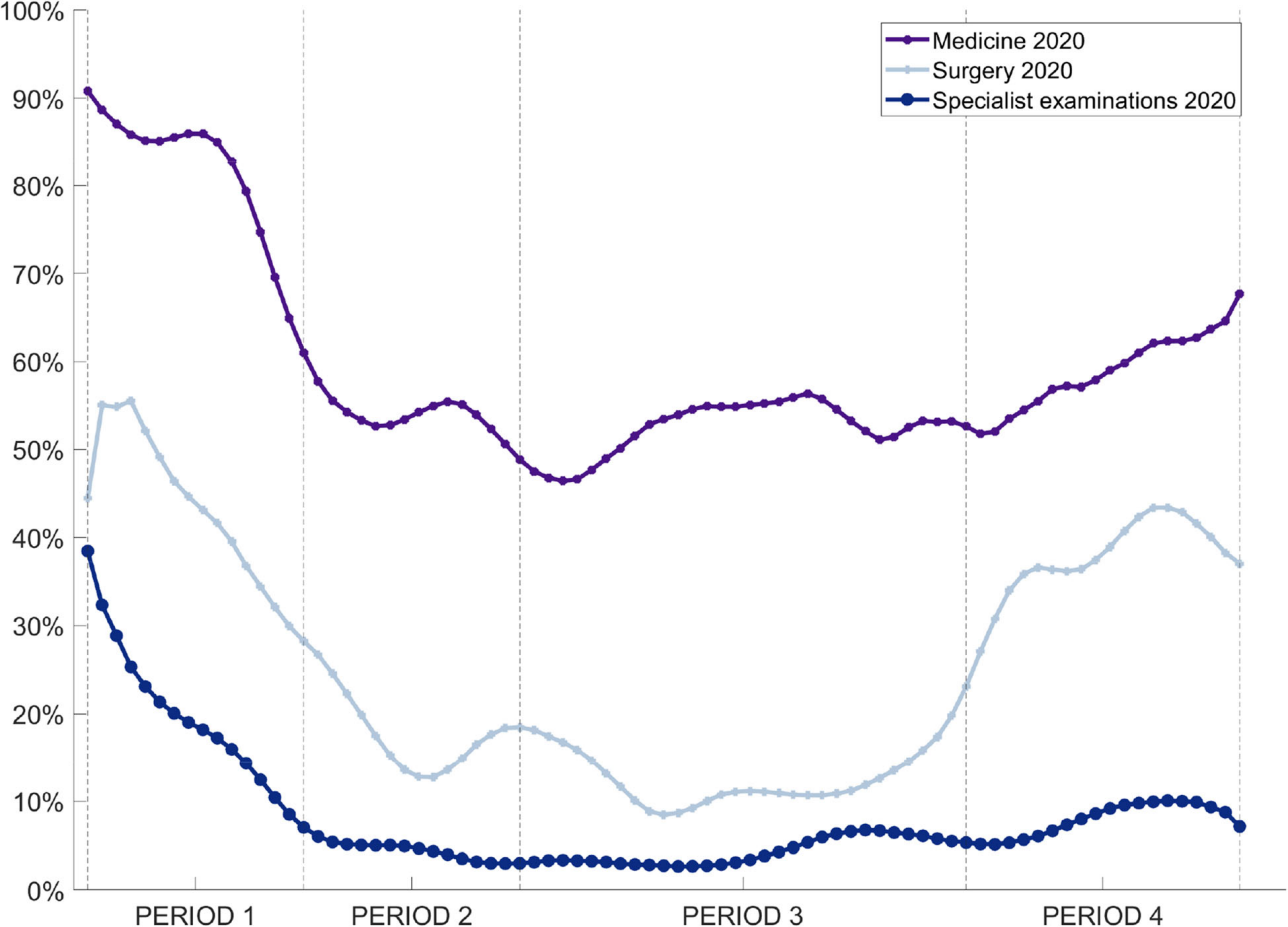
Daily ED accesses for medicine, surgery and specialist examinations in 2020, expressed as a percentage of the accesses recorded for the same time periods in 2019

In the fourth period, the drop showed a moderate reduction for medicine (40%) and surgery (63%), but it was still significant (Fig. 2).

Attendances for domestic violence exhibited a mean decrease of 86 and 85% in Periods 2 and 3, respectively, and only a 10% decrease in the fourth period compared to 2019.

Emergency psychiatric examinations decreased by 50 and 74% in the second and third period, and the trend went on in the fourth period (65%).

In 2020, deaths in ED increased by 50, 280 and 238% through Periods 1–3, and slightly reduced to 167% in Period 4, as compared to 2019.

The percentage of patients who left the ED without being seen by a physician, or during the diagnostic process (LWBS), with respect to all the ED attendances, showed a significant drop in Period 2 (2.8% vs 9.6%), and in Period 3 (5.1% vs 7.8%), while in the first period (7.2% vs 8%), and in the fourth period (6.2% vs 7.8%) the drops, though statistically significant, were less evident (Table 3).

**Table 3:** Absolute numbers and percentages for LWBS in 2019 and in 2020, with percentage of drops in 2020 compared to 2019

Period	2019			2020				
	LWBS (N)	All accesses (N)	%	LWBS (N)	All accesses (N)	%	% drop ± SE	***p*** value
1	250	3108	8	125	1715	7.2	52 ± 5.96	< 0.001
2	265	2761	9.6	22	776	2.8	92 ± 0.43	< 0.001
3	473	6030	7.8	83	1616	5.1	82 ± 1.02	< 0.001
4	277	3565	7.8	81	1308	6.2	69 ± 1.22	< 0.001

Additional file 1 in Supplemental material shows in details the daily drop for ED attendances according to specialties.

## Discussion

This study showed a significant decrease in the overall volume of ED attendances immediately before and during the COVID-19 lockdown compared to the same time-interval in 2019. The drop was significant for surgical patients, all specialist complaints, psychiatry and cases of domestic violence.

### General ED attendances

Admittances to the ED for medical complaints decreased, but less than surgical diseases, due to the high influx of severe COVID-19 patients. Correspondingly, a mean increase in mortality of 196% occurred, with a peak in the second period, when Italy had the peak of infections and of hospital and ICU admissions for COVID-19.

ED attendances reduction, although more contained, was also reported in the UK, with a 25% fall in visits during –not before- the first week following lockdown [7].

A frequently cited index of ED functioning, i.e. the percentage of patients who leave the ED without being seen by a physician, or during the diagnostic process [8, 9], showed a decrease for all four time periods examined. To our knowledge, there are no published data available regarding LWBS during mass casualty events such as pandemics. In our ED, the marked reduction compared to a previous “normal” year clearly demonstrates that people seeking care had severe conditions which were not amenable to treatment outside the hospital. For all the people who do not ultimately receive medical advice, probably for minor complaints, faster alternative tracks should be implemented during pandemics. Possibly, ad hoc facilities established outside of hospitals should be considered.

There are many aspects of the present study which need explanation. For example, in our ED the trend in visit reduction began when it seemed there were no infections in Milan, with 56 and 81% reductions in accesses for surgical and specialist examinations. In general, it is likely that people were concerned about contracting COVID-19 in hospital. In particular, ED are often crowded, and this situation was further emphasized by the media during all the pandemic. It is likely that many people experiencing mild symptoms may have decided that an ED would be a dangerous and unsafe place for non-COVID-19 patients. This is the case for admissions due to ophthalmologic, otolaryngological, urologic and orthopedic diseases. The reduction in attendances inversely corresponded with the increasing trend of SARS-CoV-2 infections.

### ED attendance for trauma

The drop in minor traumatic injuries caused by traffic collisions is easily attributable to home confinement and the drastic reductions in vehicle traffic, due to the lockdown started on March 8. Vehicle circulation was only allowed for essential workers or for serious emergency reasons. Auto-certification was required and heavy fines were imposed in cases of false declarations. On March 17, the Lombardy Government declared an overall 60% decrease in social mobility in the region [10].

For accidents at work, generally trauma falls, the drop could be due to the closures of building sites and factories. Less or no contact between people due to closure of places of socialization, with reduced alcohol and drug use, can explain the reduced attendances for stab wounds due to street crimes like assault and robbery. Such decrease was also observed in the United States [11]. However, this phenomenon should have been balanced by domestic violence, caused by home confinement of abused women and children, as it was reported in France [12] and the U.S. [13], but this was not the case in our hospital. It is possible these cases will emerge when it is easier for people to move and seek help, as evidenced by the increase in ED attendances during Period 4.

### ED attendance for surgery

What about emergency surgical cases -infections, ischemia and obstruction- which must be promptly addressed? The impact of the COVID-19 outbreak on emergency surgery in Policlinico Hospital has been significant: compared to the same time periods in 2019, surgical interventions had a 60% drop in 2020. This decrease can only partially be explained by the redistribution of cases in hospital hub designed by the Lombardy Emergency Task Force, and dedicated to time-dependent diseases -trauma, cerebrovascular accidents, coronary disease. Ideally, ED accesses for urgent and emergency conditions, such as abdominal infections, obstruction and ischemia should have remained nearly unchanged. Conversely, emergency surgery for infections, such as appendicitis, cholecystitis and diverticulitis, was performed for 14 patients, compared to 24 surgeries performed in 2019. This represents a 42% reduction. We did not observe a delay in presentation, as reported by a recent survey performed by a questionnaire sent to Italian hospitals practicing emergency surgery [14].

Reasons for reduced hospital access for infectious surgical disease remains to be explained. We can speculate that people were not directed to the ED and instead were treated conservatively, i.e. with antibiotics, by their general practitioners. If this is the case, and it can be confirmed from future statistics, the medical community should revaluate the role of out-of-hospital medical therapies in treating diseases traditionally considered of surgical interest. In-hospital non-operative management (NOM) has been suggested in recent literature for uncomplicated appendicitis, selected cholecystitis patients and colonic diverticulitis [15–21]. In the COVID-19 era, this strategy was suggested by many international recommendations [22–24] and has many advantages, first of all protecting patients and staff from possible intrahospital and in theatre virus transmission. Furthermore, NOM can save human resources and devices, and can allow ICU beds to be available. Moreover, recent literature reported an unexpectedly high rate of postoperative complications and mortality even after elective surgery [25, 26],for both infected but asymptomatic patients before surgery and patients who contracted COVID-19 after surgery [27].

Bowel and gastro-duodenal perforations halved in 2020, with 4 interventions in 2020 vs 8 interventions in 2019. Surgery for incarcerated umbilical, inguinal and incisional hernia dropped to only one case in 2020, compared to 10 cases in 2019. We suppose that home-confinement, accompanied by reductions in physical activity and hard work, may have contributed to the reduced surgeries for incarcerated hernia. However, in Spain, an increase in surgery for incarcerated hernia was reported [28], and we know that containment measures as self-isolation and home confinement were introduced in Spain as in Italy.

For gastro-intestinal perforations, we cannot identify a convincing and plausible explanation. Covered duodenal perforations, which can be treated conservatively with nasogastric suction and antibiotics, usually represent a minority of observed cases, and it seems inconsistent that such patients could stay at home and be cured only by fasting. The same for colon perforation, often caused by a complicated cancer. Obstruction, another frequent complication of colon cancer, was an uncommon finding during COVID-19 outbreak. In one case endoscopic placement of an endoprosthesis allowed surgery to be postponed for 2 weeks.

The number of surgeries for small bowel obstruction did not significantly differ between 2019 and 2020. Conservative treatment, with naso-gastric suction, *nil* *per os* and intravenous fluids, was the treatment of choice for no more than 72 h, as suggested by literature [29], and it is usually performed in the Emergency Surgery Department of Policlinico Hospital. Surgery was performed when an obstruction did not resolve or when contrast medium computed tomography showed a need for intervention without delay.

Severe COVID-19 has been associated with a marked inflammatory and prothrombotic state [30]. However no cases of mesenteric ischemia were diagnosed and operated on in COVID-19 patients during the outbreak.

Overall, the results of this study confirm a decrease in ED attendances for all medical and surgical complaints during the COVID-19 pandemic. The decrease in surgical patients was impressive and this led to a significant reduction in emergency surgeries. It is possible that some diseases were managed conservatively. However, if our data are confirmed in other settings, it is important to determine what consequences were incurred for the patients who did not seek medical attention, as well as the possible future rebound on the health care system.

### Limitations

We must acknowledge some limitations of the present work. The study did not analytically analyzed the single diagnoses of ED access to assess which diseases were missing with respect to the previous year.

Moreover, no updated data regarding surgeries performed at other hospitals in the metropolitan area of Milan are available; however, informal communications between general and emergency surgeons confirm that a drop in emergency surgeries was observed.

Finally, patients may have died at home due to complications of untreated surgical urgencies. However, it is currently difficult to assess possible changes in mortality rates for non-COVID-19 diseases from national and regional death registries.

## Conclusions

During the COVID-19 outbreak, indications for emergency surgery did not change with respect to previous periods. However, the volume of patients accessing to ED, and of surgeries for urgent and emergent diseases, did change. Actually, it is not known if the mortality rate of people who did not seek care for fear of contracting COVID-19 has risen during the pandemic. It is also unclear if, in the near future, the impressive drop in the volume of ED attendances in the COVID period will result in a rebound of more severe diseases left untreated in their early onset. Further studies, at both regional and national levels are needed to understand if drops in ED attendances and emergency surgeries during the COVID-19 pandemic will result in an increased mortality rate and unwanted complications in the population.

## Supplementary Information

**Additional file 1.** Time-series plots (with dashed trend lines) of daily ED accesses for specialities, and deaths during the same four periods in 2020 (red) and 2019 (blue).

## Data Availability

Data that support the findings of this study are available from the corresponding author upon reasonable request.

## Abbreviations

COVID-19: Coronavirus disease 2019
ED: Emergency Department
LWBS: Patients who Left the ED Without Being Seen
WHO: World Health Organization
SARS-CoV-2: Severe Acute Respiratory Syndrome-related CoronaVirus-2
NOM: Non-Operative Management
