# Supplementary material for: Variations in volume of emergency surgeries and emergency department access at a third level hospital in Milan, Lombardy, during the COVID-19 outbreak
Source: BMC Emerg Med. 2021 May 10;21:59. doi: 10.1186/s12873-021-00445-z (PMC8107771; doi:10.1186/s12873-021-00445-z)
Supplement: Supplementary file 1 — Additional file 1. Time-series plots (with dashed trend lines) of daily ED accesses for specialities, and deaths during the same four periods in 2020 (red) and 2019 (blue). [file 12873_2021_445_MOESM1_ESM.doc]

SUPPLEMENTAL MATERIAL


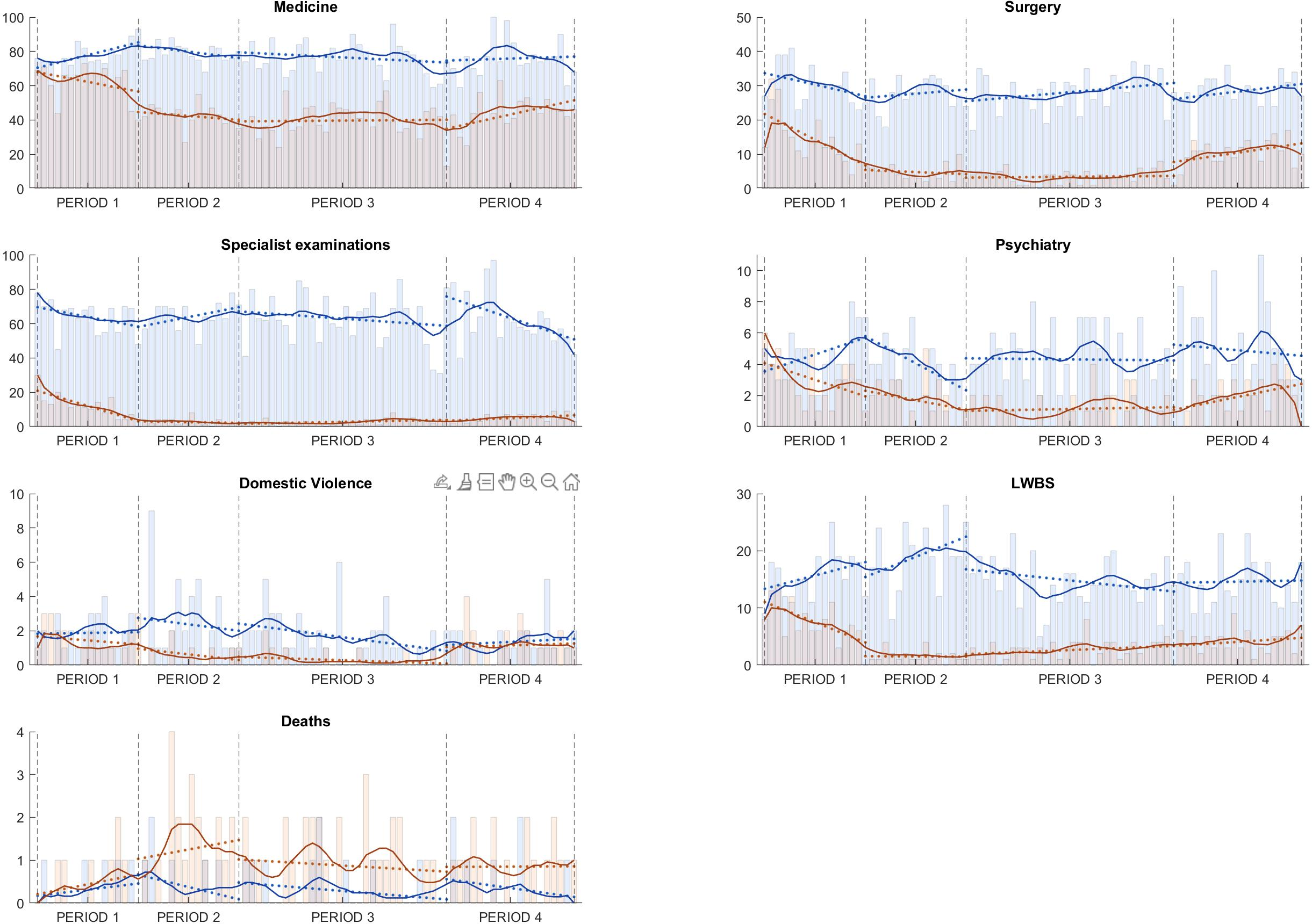


Time-series plots (and dashed trend lines) of daily ED accesses for subspecialities, and of deaths, in the four 2020 periods (red), compared to the same periods in 2019 (blue).
